# Measurement of adherence in a randomised controlled trial of a complex intervention: supported self-management for adults with learning disability and type 2 diabetes

**DOI:** 10.1186/s12874-016-0236-x

**Published:** 2016-10-06

**Authors:** Liz Graham, Judy Wright, Rebecca Walwyn, Amy M. Russell, Louise Bryant, Amanda Farrin, Allan House

**Affiliations:** 1 Leeds Institute of Clinical Trials Research, University of Leeds, Leeds, UK; 2 Leeds Institute of Health Sciences, University of Leeds, Leeds, UK

**Keywords:** Learning disability, Type 2 diabetes, Self-management, Complex interventions, Adherence, Fidelity, Reporting standards

## Abstract

**Background:**

Reporting adherence to intervention delivery and uptake is a detailed way of describing what was *actually delivered and received*, in comparison to what was *intended.* Measuring and reporting adherence is not routinely done well in complex interventions. The OK Diabetes trial (ISRCTN41897033) aimed to develop and subsequently test the feasibility of implementing a supported self-management intervention in adults with a learning disability and type 2 diabetes. A key study objective was to develop a measure of adherence to the intervention.

**Methods:**

We conducted a systematic review of published literature, extracting data from included papers using a standardised proforma. We undertook a narrative synthesis of papers to determine the form and content of methods for adherence measurement for self-management interventions in this population that had already been developed. We used the framework and data extraction form developed for the review as the basis for an adherence measurement tool that we applied in the OK Diabetes trial.

**Results:**

The literature review found variability in the quality and content of adherence measurement and reporting, with no standardised approach. We were able to develop an adherence measure based upon the review, and populate it with data collected during the OK Diabetes trial. The adherence tool proved satisfactory for recording and measuring adherence in the trial.

**Conclusion:**

There remains a need for a standardised approach to adherence measurement in the field of complex interventions. We have shown that it is possible to produce a simple, feasible measure for assessing adherence in the OK Diabetes trial.

**Electronic supplementary material:**

The online version of this article (doi:10.1186/s12874-016-0236-x) contains supplementary material, which is available to authorized users.

## Background

It is well-established that measurement and reporting of adherence to interventions is important for the transparency of recording research findings and the attribution of mechanisms of effectiveness in trials (CONSORT [1], MRC [2], NICE [3], the WIDER [4] group of journal editors, TIDieR guidance [5]). There are three main steps to ensuring that adherence is indeed dealt with properly in trials – *defining* the intervention, *measuring* and comprehensively *reporting* adherence to that intervention.

### Defining the intervention – a pre-requisite to measuring adherence

Measuring adherence is especially challenging in complex interventions, that is interventions with several interacting components [2] where more elements are at work than apply to a simple drug treatment. It is not always possible to define precisely these individual components, but rather a complex intervention can be seen to have standard ‘steps’ that must be followed [6]. Measuring adherence becomes more difficult when there are multiple components to monitor, especially where interventions are also influenced by context [7] (such as therapist characteristics) and may not be highly structured, for example allowing for individual tailoring of content. This means that careful description of the intervention is a prerequisite for establishing adherence and that the problem of understanding adherence to complex interventions is made more difficult when the component parts of such interventions are ill-defined. For example a recent systematic review of reporting standards in surgery [8] found that surgical interventions were poorly described - they were often limited to one sentence and did not allow replicability of techniques. Similarly Hoffmann, Erueti and Glasziou [9] concluded that fewer than 40 % of non-pharmaceutical trial interventions are adequately described.

### Measuring adherence

Once the intervention is defined then measuring adherence to intervention delivery and uptake is a detailed way of monitoring what was *actually delivered and received*, in comparison to what was *intended*; it is not done well in complex interventions [10]. A review of treatment adherence across 10 years of behavioural change research by Borelli et al. [11] endorsed this view, reporting that only 35 % studies used an intervention manual, 22 % provided supervision for treatment providers, and 27 % checked adherence to protocol (only 12 % used all 3, 54 % used none).

### Reporting adherence

Even for reasonably well-defined and less complex interventions, where description is relatively straightforward, there is limited *reporting* of adherence. For example, a review of problem-solving therapies found that half the included studies reported no definition or measure of adherence (Gellis and Kenaley [12]). This state of affairs led one commentary to note that “lack of change in treatment integrity practices across time and disciplines leaves the impression that treatment integrity is much like the weather - everyone talks about it, but no one actually does much about it.” [13]. Cook, Douet and Boutron [14] note that methodological research is still needed into how interventions should best be reported.

Although there is much literature detailing the poor state of adherence monitoring and reporting, there have been some developments in particular fields, for example substance abuse [15] and health behaviour change [16], as well as the development of a conceptual framework for implementation fidelity [17]. There have been reviews of adherence methodology [18], and standards for measurement of adherence have started to be included in intervention guidelines across some fields such as health behaviour change [19]. Both the CONSORT statement for non-pharmacologic treatment interventions [1] and the TIDieR checklist [5] elaborate on intervention description, including the need for adherence reporting.

The current situation, however, remains unsatisfactory: there is no uniform or transferable guidance, method or framework for ensuring comprehensive measurement of adherence and, while theoretical frameworks exist, there is little in the way of practical measurement tools or a consensus on best practice. Available frameworks tend to be too context-specific, and can be cumbersome for translation to practical application. For example, Borrelli’s treatment fidelity framework [16] is helpful in describing all elements that should be included in reports of behaviour change interventions, but the authors themselves note that “future work needs to focus not only on implementing treatment fidelity plans but also on quantifying the evaluations performed, developing specific criteria for interpretation of the findings, and establishing best practices of treatment fidelity” [20]. Similarly Carroll et al. [17] note that their conceptualisation of adherence offers a potential framework for implementation research, but that empirical work is needed to test the framework in practice.

### Developing a study-specific adherence measure

This paper reports on the development of an adherence measure for the OK Diabetes trial [21] (ISRCTN41897033), a two phase feasibility study evaluating supported self-management for adults with a learning disability and type 2 diabetes, recruited via NHS primary and secondary care as well as through the third sector. The first phase involved the development of a supported self-management intervention manual, materials and accompanying adherence measure in parallel to case finding work to identify potential participants for involvement in phase two. Phase two was a feasibility Randomised Controlled Trial (RCT) assessing the feasibility of delivering the intervention vs. usual care in people with learning disability and type 2 diabetes. A key objective was to develop an adherence measure, alongside other feasibility objectives to inform a definitive trial.

Self-management materials were developed from existing literature in learning disability and diabetes, and chronic disease self-management, and from the content of related care pathways such as that for obesity in learning disability. The intervention had selectable components to allow variable involvement with a supporter. It was delivered by diabetes nurse specialists in participants’ homes. At these nurse visits, participants were also given homework tasks to complete between sessions related to improving elements of their lifestyle which might positively impact on their diabetes. The intervention was thus tailored to each participant.

Self-management interventions have no gold standard definition but all include active involvement of the patient to manage their condition and associated behaviours outside the routine clinical setting [22]. Thus methods to monitor adherence to a self-management intervention need further consideration as existing frameworks largely focus on therapist-client interactions as a means of assessing adherence, whereas we needed to find a way of assessing adherence not only to session attendance and content delivery, but also to use of self-management techniques (the active component of the intervention) between times. The exact methods of doing so were not clear, particularly since existing frameworks suggest observation of intervention delivery and participant enactment, or participant reporting of skills learned, neither of which would have been practical for this intervention (individually delivered at home with ongoing integration in daily living) and population (people with a learning disability). We found no existing *generic* framework or guidance that could be applied to the objective measurement of adherence to treatment in this study.

Our primary objective in relation to intervention adherence was to develop a *measure* to capture both provider and participant adherence to the elements of the developed intervention and which generated an output that allowed simple *reporting.* Although our main objective was to develop an adherence measure for our own study, by summarising the current methods used we hope to describe a tool not just for use in the OK Diabetes study but also to contribute to the development of a standardised approach to measuring adherence that would have wider applicability in other complex interventions and especially those involving an element of self-management.

## Methods

We conducted a review of the literature to determine the methods of adherence measurement reported to date for similar interventions (self-care, self-management) in the same population (learning disability), to see if there were helpful techniques we could adopt.

### Search strategy

We ran literature searches in July 2013, and update searches in July 2015, on the following databases: EBSCO Cumulative Index to Nursing and Allied Health Literature (1980 to 2015 July 15), Ovid Embase Classic + Embase (1947 to 2015 July 13), Ovid MEDLINE(R) 1946 to July Week 1 2015, Ovid MEDLINE(R) In-Process & Other Non-Indexed Citations July 14 2015, Ovid PsycINFO 1806 to July week 1 2015.

Search strategies were developed for five concepts: learning disability/ies, self-management interventions, type 2 diabetes or weight loss, adherence (including compliance) and evaluative studies. The combination of terms allowed identification of 1) studies describing measures of adherence to diabetes self-management or weight-loss self-management interventions for people with learning disabilities, and 2) evaluative studies of self-management interventions for managing diabetes or weight in people with learning disabilities. These evaluative studies were included in case they described measures of adherence using terms we may have inadvertently missed in our descriptors of ‘adherence’. The search strategies aimed at a high specificity rather than high sensitivity to identify key papers in the field. To ensure further key papers were identified we searched for secondary citations in identified papers, and for included papers we tracked citations forwards using Science Citation Index and Google Scholar. Full search strategies for each database searched can be found in Additional file 1.

### Inclusion and exclusion criteria

Papers were included if they:

- described primary research studies;
- involved participants who were adults with a learning disability;
- described a standardised self-management or self-care intervention;
- described an intervention aimed at weight loss or improving self-management of diabetes.

We defined self-management as involving at least (i) some definition of actions relevant to improving the participants condition (overweight or diabetes) (ii) setting and recording of specific goals or targets related to those actions (iii) monitoring of progress in achieving those goals. The person with a learning disability had to be an active agent in this process – albeit with the help of a supporter at times – so that they were not just a passive recipient of a programme designed and delivered by a third party.

Exclusion criteria included:

- participants were children, or adults with dementia, as these were not relevant to our study population;
- the intervention had no active self-management component e.g. attendance at a structured gym class arranged by residential care staff;
- studies using qualitative methods only;
- literature reviews;
- no available English version.

### Study selection

An initial screening of all titles and abstracts against the inclusion and exclusion criteria was undertaken by AH and LG in order to identify potentially relevant papers. For studies that appeared to meet the inclusion criteria, or where there was uncertainty, full papers were requested and reviewed in detail by LG. AH reviewed all abstracts and a random sub-sample of 20 % papers. When a level of uncertainty remained regarding relevance, AH also reviewed those papers and consensus was reached regarding appropriateness for inclusion.

Papers were not quality assessed against a checklist or checked for risk of bias because we were interested in the form and content of adherence measurement and reporting, which we determined regardless of study quality.

### Developing a framework for data extraction

In a series of research team meetings we developed an initial framework for data extraction based upon our reading of the wider adherence literature as well as the literature identified in the present search. We distinguished:

- Steps taken to *ensure* the intervention was delivered correctly in form, content and quality
- Steps taken to *measure for research* that these approaches to ensuring quality of delivery were employed
- Steps taken to *measure* actual provider adherence
- Steps taken to *measure* participant adherence

The first two steps fit with existing literature describing ‘fidelity’ - the degree to which provider delivery is in line with the intended form and content. The latter two steps fit with the existing definition of ‘adherence’.

To ensure a measure of adherence was applied to all elements of the intervention not just a selected few, our initial framework had four categories to describe an intervention:

- The content of the intervention - topics or components covered, such as (for diabetes self-management) shopping for and preparing food, planning physical activity, taking tablets, and avoiding unhealthy behaviours like smoking or drinking too much alcohol.
- The techniques employed in the intervention – how it is delivered, for example through education, training in goal setting, use of self-monitoring and feedback techniques.
- The platform or format by which it is delivered – for example written materials, group sessions, self-completion charts, web-based resources, text messaging.
- The degree of individualisation of the intervention. This could mean use of inclusion and exclusion criteria to define the sample from the target population to whom the intervention is delivered, or modification of elements of the intervention to suit the needs of individuals within that sample - for example those with visual impairments.

We used this framework as the basis for a data extraction form which, after field-testing and minor adaption, we found suitable over the course of reviewing the identified papers. Where changes were made to the data extraction form, papers were reviewed again to refine the extracted items in line with the revised extraction process. The final data extraction form included detail relating to the type of study participants, type of intervention(s), content of the intervention, how the intervention was delivered, how both provider and participant adherence were measured, collected and scored, and how quality and competence were ensured. For simplicity we combined the ‘techniques employed’ domain and ‘delivery format’ domain into a single domain describing practical delivery details. Because all the studies included individualisation of materials to allow use by adults with learning disability we did not use this field for further data extraction.

Table 1 summarises the elements considered during data extraction. The full form can be found at Additional file 2, with a summarised version in the form of a usable checklist at Additional file 3.

**Table 1:** Elements considered during data extraction

Intervention	Was this described?	Adherence measurement	
		Provider	Participant
Content (topics, components)	Y/N	Was there measurement of the degree to which these topics were covered?	Was there measurement of what the participant received about these topics?
Practical delivery details (techniques used, format of delivery, contact)	Y/N	Was there measurement of what materials, sessions, training, etc. were provided, and whether they followed the prescribed format?	Was there measurement of sessions attended, techniques used, etc.?
Intervention quality/competence of delivery	Y/N	Were credentials, competency, training and supervision measured?	

### Data synthesis

As there were so few data available regarding treatment adherence in this population and because they were so heterogeneous, there was no possibility of data pooling. We thus undertook a narrative synthesis, organised according to the framework described above, to explore the approaches to measuring adherence in the included studies. We looked for any similarity between studies’ approaches to measuring and reporting adherence to the described intervention, and documented descriptions of the range of approaches and their robustness. The intention was to develop the data extraction form into a more usable tool for measuring adherence in the OK Diabetes trial.

### Developing an adherence measure based upon the data extraction form

Our supported self-management intervention had several components to it, used as a basis to *describe* the intervention: [1] identifying the participants’ daily routines, activity and eating habits, [2] identifying aspects of diabetes management, [3] identifying key supporters and helpers, [4] setting goals for change that involved supporters where possible, [5] monitoring progress. For each component of the intervention a record was generated either by the nurse (for example, a list of supporters, identified goals) or by the person with diabetes (for example, a note of goal-focussed activity undertaken).

We identified which of these records we could collect routinely for each participant, using the framework of our data extraction form to structure these elements of the intervention to ensure all aspects of adherence were being recorded. To *measure* adherence we also developed a scoring system based upon completion rates, which would allow us to estimate the proportion of completed tasks during the supported self-management intervention, including an overall judgement about whether the whole process had been completed at least once.

Finally, we planned to develop a summary Case Report Form (CRF) to enable collation of these adherence items and the adherence score to facilitate *reporting*.

## Results

The electronic searches identified 573 references, which were reduced to 464 after duplicates were removed.

Following review of the 464 abstracts identified from the electronic searches, 22 were identified as possible studies for review, and the full papers obtained. Review of these papers excluded a further 10 (literature reviews which did not report adherence measurement, non-interventional qualitative studies, physical activity classes only, conference or thesis abstracts with no further detail available, one paper not available in English) leaving 12 for detailed review of the adherence measurement approaches adopted for the reported intervention(s).

Scrutiny of the reference lists of the 22 papers initially identified for full review revealed a further 32 studies. Of these 14 were excluded (did not include self-care intervention, formal physical activity classes only, not available online or via the British Library) and 18 were reviewed in detail.

**Fig. 1:**
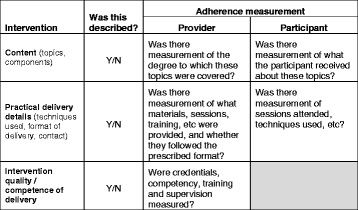
Studies identified and reasons for inclusion and exclusion

Figure 1 details the number of studies identified and reasons for exclusion and inclusion at each stage.

Additional file 4 provides an overview of the 30 studies reviewed [23–52], based on the elements of the intervention described, and provider and participant adherence reported, as illustrated in Table 1.

Table 2 summarises the intervention and adherence elements found in the studies reviewed.

**Table 2:** Intervention and adherence elements reported in reviewed studies

Intervention elements described	Details reported in papers reviewed (*N* = number of studies where details provided, total *N* = 30)						
Content	Choosing the right foods (*N* = 21)	Preparing food (*N* = 9)	Eating behaviours (*N* = 12)	Physical activity – formal (*N* = 7) and informal (*N* = 19)	Behaviour modification (*N* = 7)	Health awareness/health assessment/self-care (*N* = 8)	
Delivery technique	Formal education (*N* = 24)	Goal setting (*N* = 9)	Action plans (*N* = 7)	Skills training (*N* = 8)			
Method of delivery	Group sessions (*N* = 26)	Individual sessions (*N* = 4)	Provision of educational materials (*N* = 12)	Participants trained in use of self-monitoring resources (*N* = 4)			
Formal Measurement described:							
- Steps to ensure quality of provider	Selection of providers based on experience (*N* = 7)	Training in the intervention (*N* = 10)	Supervision/feedback (*N* = 2)	Standard script to guide intervention sessions (*N* = 1)	Intervention guidance (*N* = 1)		
- Delivery by provider (content and quality)	Scoring system to assess content delivered (*N* = 2)	Observation or recording of session content (*N* = 3)	Materials provided (*N* = 2)	Interviews regarding delivery (*N* = 1)	Provider attrition (*N* = 1)	Provider meetings (*N* = 1)	Recommendations after intervention delivery (*N* = 1)
- Participant adherence	Attendance at/duration of sessions reported (*N* = 9)	‘Completers’ reported (*N* = 12)	Homework completion reported (*N* = 3)				
Adherence included in analysis & reporting	Described completers vs. non-completers (*N* = 6)	Outcomes split by attendance rates (*N* = 4)	Reported adherence score (*N* = 1)	Reported outcomes in relation to adherence (*N* = 1)			

Two thirds of the studies included participants with learning disabilities only, while the remaining third also included members of staff in community residences, clinics or carers to assist with the intervention.

Every study had at least one intervention component that required provider adherence and one that required participant adherence: there was thus the research opportunity for adherence to these components to be measured and reported. For example, the following could have been measured for providers - training in and supervision of the delivery of the intervention, attendance at sessions and delivery of prescribed content in each session, provision of materials; and the following could have been measured for participants - attendance at sessions, use of materials, completion of homework diaries, and so on.

However, we found patchy descriptions of how adherence to each intervention was measured. Some studies identified some (but not all) components of the intervention delivery and receipt processes that were measured, whilst others described only who delivered the sessions, participant attendance rates and nothing further. One study did not report any measure of adherence, 11 studies reported no detail relating to provider adherence beyond number of sessions provided, and one study provided no information about participant adherence.

Study intervention components and delivery were generally reported well. We used our data extraction form to determine what elements of adherence could be identified from the papers reviewed. All studies had a degree of intervention description but there was little detail provided for some.

Content: Intervention content was typically well reported and largely focussed on choosing the ‘right’ foods, eating behaviours and increasing physical activity.

Techniques: were invariably described. The majority (24/30) delivered their intervention via formal education. A number of studies utilised goal setting (9/30), making action plans (7/30) and skills training (8/30).

Delivery: Education was delivered face-to-face in group sessions, through provision of written educational materials (13/30) for subjects to read or view, or in some cases participants were trained in the use of diaries and keeping records of newly-acquired behaviours or activities.

Individualisation: All studies used the presence of learning disability as an inclusion criterion, and all interventions were designed (or individualised) for this group.

Steps to ensure provider compliance to content, format and quality were rarely reported. The most frequently reported quality assurance steps were training in the intervention techniques (10/30), and selection of providers based on prior experience or qualifications (7/30). There was limited reporting of the use of supervision or feedback (2/30), a script to guide the intervention sessions (1/30), and intervention guidance (1/30).

Measurement of steps to ensure content and quality of delivery was rarely reported. In only two papers [25, 36] was there report of a specific adherence scoring system to assess content covered in intervention sessions, and in only three [26, 31, 45] was there mention of observation or recording of session content for review. Others reported that intervention materials had been provided (2/30), that interviews regarding delivery were conducted (1/30), that process notes were made during delivery (1/30), provider attrition rates (1/30), provider meeting frequency (1/30), and that recommendations were made following intervention delivery (1/30). Although there is some level of measurement here, it is not sufficiently comprehensive to fully describe or understand provider adherence.

Research measurement of provider adherence such as ‘provision of sessions’ and ‘competence of providers’ were often included in the methods section of articles, but data recording that sessions were *actually* delivered and that competence was *formally measured* were not found in any study.

Participant Adherence was rarely measured in a formal way. Some studies described that attendance was monitored but did not report how this was captured or any data pertaining to attendance. A number of studies *described* the methods they could have or did employ to measure certain aspects of adherence to the intervention, but failed to *report* these in the results section. For example 12 studies noted use of homework, diaries or logs between sessions, but only 3 *reported* any data regarding homework completion. Other studies reported adherence (e.g. % attendance) without explaining the methods used to capture the data.

Where participant adherence was reported, this focused mainly on attendance at sessions (9/30), or retention at follow up or ‘completers’ (12/30). Very little detail was provided about participants’ successful completion of required steps. Adherence to any of the described processes was only reported in 3 studies.

Utilisation of adherence measures to explain or discuss outcomes was sparse. Where adherence measures were incorporated into analyses, these largely served to either exclude non-completers or to split results into those of completers and non-completers (6/30), or to split outcomes in relation to attendance rates (4/30). Only one study [36] reported the use of each component of the intervention in relation to an adherence score, and noted that modifications would be made to the intervention based on adherence score analysis. One study [25] looked at outcomes in relation to adherence.

### A tool for the OK diabetes study

Our data extraction tool, the comprehensiveness and feasibility of which was tested during review of the papers, informed the development of a suite of Case Report Forms (CRFs) for use in the OK Diabetes study.

These CRFs incorporated the elements of intervention fidelity, delivery and receipt that could be measured for the OK Diabetes study. One CRF was designed for completion by the study nurse delivering the intervention (to record attendance), one by an independent reviewer (who looked at materials in case files to ascertain delivery of specific elements of the intervention as well as receipt by the participant evidenced by return of ‘calendar sheets’ to indicate completion of a particular activity), and one by the supervisor (to evidence regular attendance at supervision as per intervention protocol). A further CRF was designed for completion independently at the end of a participant’s involvement which summated all elements of delivery to give an overall ‘adherence score’ for that participant: this included the proportion of completed tasks and an overall judgement about whether the essential components of a whole intervention cycle (review of lifestyle factors, identification of goals and enactment of those goals) had been completed at least once, which was our definition of acceptable or adequate adherence. CRFs can be found in Additional file 5.

During the conduct of the OK Diabetes trial we were able to use these CRFs successfully to record adherence and derive an adherence score. Of the 82 people randomised, follow up data and primary outcome were obtained in 77 (94 %) and we were able to measure adherence and derive an adherence score using our measure in all these cases. Details of the OK Diabetes trial and its findings will be reported elsewhere.

## Discussion

Our review found that adherence to self-management interventions for diabetes or weight loss in people with a learning disability was poorly measured and reported, as it is in other settings. We found no consistency of approach to adherence measurement despite there being overlap in terms of intervention content, delivery and target population. Elements that could have been reported as clear indicators of adherence were often missing. For those studies in our review where adherence was measured, there was a range of sophistication in standards of reporting - from none, to minimal descriptive reporting of provider training and participant attendance, to reporting of results for a study-specific adherence scoring system in two cases [25, 36].

We developed a data extraction tool for describing the elements of adherence, and used it to extract data from existing literature; we subsequently used it to inform the development of a project-specific adherence measurement approach for the OK Diabetes trial. Although our tool is derived from specialist literature, we found it compatible with two other comprehensive frameworks we identified [16, 17] with the advantage of greater simplicity. We wanted an approach that would [1] work for self-management (where it is not possible to observe adherence), [2] work for people with a learning disability who cannot always provide complex self-report of activities, and [3] be very simple, to facilitate it’s use in low resource settings. Our framework included elements relating to content, delivery and exposure, in line with Borrelli and Carole’s frameworks. What it didn’t include was detail relating to dose equivalence, mechanisms of action, assessing the presence of essential and proscribed elements, and assessment of participant understanding. All these elements are either not applicable to a self-management intervention (which by its very nature is less prescriptive and more individually tailored) or not appropriate for people with a learning disability. What our tool did include was the checking of materials provided, and use of materials or techniques between intervention delivery sessions.

An adherence measurement tool should: [1] allow an overall decision to be made regarding adequacy, and quality of delivery and receipt, [2] produce an overall metric to allow judgement regarding the degree of exposure and [3] enable review of which elements of an intervention were delivered to allow assessment of feasibility of delivery and effectiveness (which elements work and which don’t work). Our tool did enable a quality assessment (1, above); and it partially achieved a reliable metric in relation to degree of exposure (2, above), although return of ‘calendar sheets’ detailing activities undertaken by participants was patchy. Enhancing compliance with participant self-reporting would be an area to focus on in future self-management intervention research in this population. We were able to assess which elements had been delivered by the nurse (feasibility), but it was not possible to identify specific components which could be said to be essential, as a self-management intervention is individually tailored and so does not rely on standard, pre-identified elements (3, above).

CONSORT [1] and TIDieR [5] guidance both recommend completeness of reporting of intervention description, implementation and adherence; however their focus is on what needs to be reported rather than what needs to be measured. The SPIRIT statement [53] provides guidance on standard items to include in an intervention trial protocol, including a section on adherence. This is currently focussed on improving adherence within drug trials (to medication regimes), and we suggest that it could be expanded to include more detail relating to complex intervention trials. Consideration of strategies to enhance adherence and on how to measure it at the stage of writing the study protocol would seem well placed.

We have generated a usable, simple, descriptive tool for our study. We believe it shows promise as a means to generate adherence data collection tools for other self-management interventions, as well as to extract data from existing literature to assess adherence measurement and reporting.

The main limitations of our study are two-fold. First, we did not contact experts in the field – either published authors or active researchers – because we were under time pressure to develop an adherence measure for use in an externally-funded RCT delivered on a pre-determined timetable. Second, we have not checked the utility of our tool beyond the specific project for which it was developed.

## Conclusions

We have shown that it is possible to produce a simple, feasible measure for assessing adherence in the OK Diabetes trial, which could be more widely applicable to other self-management interventions and which requires only modest input from the research team, those delivering the intervention and participants.

Measurement of adherence to interventions is critical to the reporting of RCTs – to allow replicability and confidence in reported outcomes. Having a robust, effective, but simple approach to adherence measurement is an attractive proposition for trialists working to tight timelines and budgets. We hope that our findings will contribute to the development of a more widely accepted approach to adherence measurement in complex interventions.

## Additional files

Additional file 1: Search Strategies by Database. This includes explanatory search notes, and details of all terms and dates used for each database searched. (DOCX 39 kb)
Additional file 2: Data Extraction Form. (DOCX 19 kb)
Additional file 3: Adherence Checklist – developed from the data extraction form. (DOCX 16 kb)
Additional file 4: Summary of Papers Reviewed. This table summarises the study design, intervention content, techniques employed, format of intervention delivery, and steps taken to ensure provider and participant adherence for all papers meeting eligibility criteria and reviewed as part of this work. (DOCX 41 kb)
Additional file 5 Case Report Forms developed to collect adherence data for the OK Diabetes Trial. (PDF 447 kb)

## Abbreviations

CONSORT: Consolidated standards of reporting trials
ISRCTN: International standard randomised controlled trial number
MRC: Medical research council
NICE: National institute for health and care excellence
RCT: Randomised controlled trial
SPIRIT: Standard protocol items: recommendations for interventional trials
TIDieR: Template for intervention description and replication
WIDER: Workgroup for intervention development and evaluation reporting
