# Supplementary material for: Measurement of adherence in a randomised controlled trial of a complex intervention: supported self-management for adults with learning disability and type 2 diabetes
Source: BMC Med Res Methodol. 2016 Oct 6;16:132. doi: 10.1186/s12874-016-0236-x (PMC5052902; doi:10.1186/s12874-016-0236-x)
Supplement: Additional file 1: — Search Strategies by Database. This includes explanatory search notes, and details of all terms and dates used for each database searched. (DOCX 39 kb) [file 12874_2016_236_MOESM1_ESM.docx]

**Search Strategies by Database**

**Searches undertaken**

**Search Notes**

‘Dementia’ term (textword and subject heading) was tested and used to remove papers regarding Diabetes linked to early dementia or Management of diabetes in dementia. This seemed successful in removing irrelevant papers whilst keeping the useful ones.

**CINAHL (EBSCO) 1981- 15-07-15**

S34 S30 NOT S33 110

S33 S31 OR S32 325,900

S32 (MH "Dementia+") OR TI Dementia OR AB dementia 40,326

S31 ((MH "Child+") OR (MH "Adolescence+")) NOT ((MH "Adult+") AND ((MH "Child+") OR (MH "Adolescence+"))) 285,894

S30 S5 AND S12 AND S20 AND S29 148

S29 S21 OR S22 OR S23 OR S24 OR S25 OR S26 OR S27 OR S28 662,745

S28 (MH "Treatment Refusal") OR MH refusal to participate 3,331

S27 ((MH "Medication Compliance") OR (MH "Patient Compliance")) OR TI ( adher* or nonadher* or non-adher*) OR TI (compliance or noncompliance or non-compliance) OR AB (adher* or nonadher* or non-adher*) OR AB ( compliance or noncompliance or non-compliance) 48,002

S26 TI (crossover* or "cross over*" or cross-over*) OR TI ((trial or trials) n2 (clinical or controlled or cluster or factorial)) OR TI case Stud* OR TI intervention OR AB (crossover* or "cross over*" or cross-over*) OR AB ((trial or trials) n2 (clinical or controlled or cluster or factorial)) OR AB case stud* OR AB intervention 190,384

S25 TI ((blind or mask*) n2 (singl* or doubl* or trebl* or tripl*)) OR TI control group* OR TI (outcome stud* or quasiexperimental or "quasi experimental" or quasi-experimental or "pseudo experimental") OR AB ( (blind or mask*) n2 (singl* or doubl* or trebl* or tripl*)) OR AB control group* OR AB (outcome stud* or quasiexperimental or "quasi experimental" or quasi-experimental or "pseudo experimental") 62,911

S24 TI (random* n3 (study or studies or trial or trials)) OR TI ( random* n3 (allocation or assign* or allocate*)) OR TI (study n1 (pilot or feasibility or evaluation or validation)) OR TI (studies n1 (pilot or feasibility or evaluation or validation)) OR AB (random* n3 (study or

Studies or trial or trials)) OR AB (random* n3 (allocation or assign* or allocate*)) OR AB (study n1 (pilot or feasibility or evaluation or validation)) OR AB (studies n1 (pilot or feasibility or evaluation or validation)) 96,882

S23 (MH "Placebos") 7,319

S22 MH "Multicenter Studies") OR (MH "Pilot Studies") OR (MH "Validation Studies") OR (MH "Evaluation Research+") OR (MH "Case Studies") OR (MH "Comparative Studies") OR (MH "Multimethod Studies") 159,971

S21 (MH "Experimental Studies+") OR (MH "Nonexperimental Studies+") OR (MH "Crossover Design") 412,142

S20 S13 OR S14 OR S15 OR S16 OR S17 OR S18 OR S19 145,076

S19 (MH "Health Promotion/MT") 4,781

S18 (MH "Assistive Technology Devices") OR TX assistive n2 technolog* 6,947

S17 TX (self manage* n5 (device* or tool* or technolog*)) OR TX ("self help" n5 (device* or tool* or technolog*)) OR TX ("self care" n5 (device* or tool* or technolog*)) 367

S16 TI (teach* n2 (Skill or skills)) OR AB (teach* n2 (Skill or skills) ) OR TI (education n2 (program* or session*)) OR AB (education n2 (program* or session*)) OR TI (patients n5 (information or leaflet* or education* or educate* or book* or pamphlet* or resource* or dvd* or cd or cds or "cd rom*" or multimedia or web* or internet*)) OR AB (patients n5 (information or leaflet* or education* or educate* or book* or pamphlet* or resource* or dvd* or cd or cds or "cd rom*" or multimedia or web* or internet*)) 31,814

S15 TI (self n3 (help or care* or manage* or administer* or monitor* or medicat* or treat* or inject*)) OR AB (self n3 (help or care* or manage* or administer* or monitor* or medicat* or treat* or inject*)) OR TI goal* n3 set* OR AB goal* n3 set* OR TI (patient n5 (information or leaflet* or education* or educate* or book* or pamphlet* or resource* or dvd* or cd or cds or "cd rom*" or multimedia or web* or internet*)) OR AB (patient n5 (information or leaflet* or education* or educate* or book* or pamphlet* or resource* or dvd* or cd or cds or "cd rom*" or multimedia or web* or internet*)) 46,423

S14 (MH "Motivation") OR (MH "Goal-Setting") 18,585

S13 ((MH "Self Care") OR (MH "Blood Glucose Self-Monitoring") OR (MH "Self Administration") OR (MH "Self Medication")) OR ((MH "Patient Education") OR (MH "Diabetes Education")) 63,648

S12 S6 OR S7 OR S8 OR S9 OR S10 OR S11 231,496

S11 (MH "Therapeutic Exercise+") 28,176

S10 TX ((diet* or eat or eating) n2 (behav* or habit* or therap* or treatment* or intervention*)) OR TX (weight n2 (loss* or control or gain* or reduc*)) OR TX (physical n2 (exercise* or activit*)) OR TI exercise* Research Databases 102,174

S9 (MH "Diet Therapy") OR (MH "Restricted Diet+") 7,829

S8 MH "Food Habits" OR MH "Exercise+" OR MH "Diet" OR MH "Obesity+" Search modes 113,523

S7 TI ((diabet* n3 (ii or "type 2" or two))) OR AB ((diabet* n3 (ii or "type 2" or two))) OR TI ((diabet* n3 ("non insulin dependent" or stable or "adult onset" or "maturity onset" or "ketosis reistant" or "slow onset"))) OR AB ((diabet* n3 ("non insulin dependent" or stable or "adult onset" or "maturity onset" or "ketosis reistant" or "slow onset")) ) OR TX (niddm or mody) 20,367

S6 (MH "Diabetes Mellitus") OR (MH "Diabetes Mellitus, Type 2") Search modes 54,494

S5 S1 OR S2 OR S3 OR S4 57,655

S4 TX (((Development* or Learning or intellectual*) n3 (disab* or disorder* or disturbance* or impair* or difficulty or difficulties))) OR TX (((Subnormal* or below normal) n3 (intellect* or mental* or learning or IQ))) OR TX ((Mental* n3 (disab* or impair* or deficien* or retard* or handicap*))) OR TX ((cogniti* n5 (disab* or disorder* or disturbance* or impair* or difficulty or difficulties))) 57,425

S3 MH cognition disorders 13,205

S2 MH intellectual disability OR MH developmental disabilities Search modes 13,570

S1 (MH "Learning Disorders") OR (MH "Reading Disorders") OR (MH "Mental Retardation+") 4,833

**Embase Classic+Embase <1947 to 2015 July 13>**

1 exp learning disorder/ (29817)

2 intellectual impairment/ (12727)

3 *cognitive defect/ (35426)

4 mental deficiency/ (68333)

5 ((Learning or intellectual*) adj3 (disab* or disorder* or disturbance* or impair* or difficulty or difficulties)).tw. (36734)

6 (development* adj1 (disab* or disorder* or disturbance* or difficulty or difficulties)).tw. (16067)

7 ((Subnormal* or below normal) adj3 (intellect* or mental* or learning or IQ)).tw. (1029)

8 (Mental* adj3 (disab* or impair* or deficien* or retard* or handicap*)).tw. (57952)

9 or/1-8 [Learning Disability] (185122)

10 diabetes mellitus/ (435354)

11 limit 10 to yr="1900 - 1980" (69920)

12 non insulin dependent diabetes mellitus/ (161012)

13 (diabet* adj3 (ii or "type 2" or two)).tw. (141851)

14 (diabet* adj3 ("non insulin dependent" or stable or "adult onset" or "maturity onset" or "ketosis reistant" or "slow onset")).tw. (15810)

15 (niddm or mody).tw. (9124)

16 ((diet* or eat or eating) adj2 (behav* or habit* or therap* or treatment* or intervention*)).tw. (57190)

17 (weight adj2 (loss* or control or gain* or reduc*)).tw. (180343)

18 (physical adj2 (exercise* or activit*)).tw. (105665)

19 exercise*.ti. (112786)

20 exp diet/ (263288)

21 eating habit/ (8279)

22 exp obesity/ (345937)

23 exp exercise/ (252203)

24 exp *diet therapy/ (73412)

25 or/11-24 [Diabetes or Diet or Exercise] (1251413)

26 self care/ or self help/ or self medication/ (55724)

27 self monitoring/ (4843)

28 (self adj3 (care* or manage* or administer* or monitor* or medicat* or treat* or inject*)).tw. (86890)

29 drug self administration/ (8535)

30 *health promotion/ (31908)

31 *patient education/ (25097)

32 (goal* adj3 set*).tw. (7314)

33 motivation/ (78246)

34 *teaching/ (33095)

35 ((patient or patients) adj5 (information or leaflet* or education* or educate* or book* or pamphlet* or resource* or dvd* or cd or cds or "cd rom*" or multimedia or web* or internet*)).tw. (152180)

36 (education adj2 (program* or session*)).tw. (31577)

37 ((skill or skills) adj2 teach*).tw. (3541)

38 (("self help" or "self care" or "self manage*") adj5 (device* or tool* or technolog*)).tw. (957)

39 or/26-38 [Self Care Interventions] (446772)

40 exp randomized controlled trial/ (379523)

41 exp double-blind procedure/ (126542)

42 exp single-blind procedure/ (20592)

43 exp crossover-procedure/ (43954)

44 ((singl* or doubl* or trebl* or tripl*) adj (blind* or mask*)).tw. (181216)

45 evaluation/ (170346)

46 exp comparative study/ or feasibility study/ or pilot study/ or validation study/ (1258639)

47 exp methodology/ (4074555)

48 placebo*.tw. (227010)

49 (random* adj3 (study or studies or trial or trials)).tw. (388271)

50 (random* adj3 (allocation or assign* or allocate*)).tw. (126332)

51 (study adj (pilot or feasibility or evaluation or validation)).tw. (1481)

52 (control* adj (group or trial* or stud* or evaluation* or experiment*)).tw. (712804)

53 ("outcome study" or "outcome studies" or quasiexperimental or "quasi experimental" or quasi-experimental or "pseudo experimental").tw. (16804)

54 ((trial or trials) adj2 (clinical or controlled or cluster or factorial)).tw. (490857)

55 case stud*.tw. (80498)

56 intervention.tw. (516290)

57 or/40-56 [Intervention Studies] (5968230)

58 patient compliance/ or dietary compliance/ or medication compliance/ (111197)

59 (adher* or nonadher* or non-adher*).tw. (181330)

60 (compliance or noncompliance or non-compliance).tw. (130503)

61 or/58-60 (354928)

62 57 or 61 [Intervention of Adherence Studies] (6175123)

63 9 and 39 and 25 and 62 (202)

64 treatment refusal/ or refusal to participate/ or patient participation/ or patient attitude/ or patient satisfaction/ or patient preference/ [additional compliance SH] (178244)

65 57 or 61 or 64 [Intervention or Adherence or compliance Studies] (6250776)

66 9 and 39 and 25 and 65 (207)

67 exp animal/ not (exp animal/ and exp human/) (4753062)

68 (exp adolescent/ or exp child/ or exp newborn/) not ((exp adolescent/ or exp child/ or exp newborn/) and (adult/ or middle aged/ or exp aged/)) (2002455)

69 dementia.tw. (102357)

70 exp dementia/ (256301)

71 or/67-70 (6870434)

72 63 not 71 [original strategy] (150)

73 66 not 71 [extra compliance terms] (155)

**Database: Ovid MEDLINE(R) <1946 to July Week 1 2015>**

1. learning disorder/

2. Mentally Disabled Persons/

3. Cognition Disorders/

4. Intellectual Disability/

5. ((Development* or Learning or intellectual*) adj3 (disab* or disorder* or disturbance* or impair* or difficulty or difficulties)).tw.

6. ((Subnormal* or below normal) adj3 (intellect* or mental* or learning or IQ)).tw.

7. (Mental* adj3 (disab* or impair* or deficien* or retard* or handicap*)).tw.

8. (cogniti* adj5 (disab* or disorder* or disturbance* or impair* or difficulty or difficulties)).tw.

9. or/1-8 [Learning Disabilities]

10. exp Diabetes Mellitus, Type 2/

11. Diabetes Mellitus/

12. (diabet* adj3 (ii or "type 2" or two)).tw.

13. (diabet* adj3 ("non insulin dependent" or stable or "adult onset" or "maturity onset" or "ketosis reistant" or "slow onset")).tw.

14. niddm.tw.

15. mody.tw.

16. or/10-15 [Diabetes Type 2]

17. exp diet/

18. nutrition therapy/

19. food habits/

20. dietetics/

21. nutritional sciences/

22. diet therapy.fs.

23. ((diet* or eat or eating) adj2 (behav* or habit* or therap* or treatment* or intervention*)).tw.

24. (weight adj2 (loss* or control or gain* or reduc*)).tw.

25. exp obesity/

26. exp weight loss/

27. exp exercise/

28. exp exercise therapy/

29. (physical adj2 (exercis* or activit*)).tw.

30. exercise*.ti.

31. or/17-30 [Diet or Exercise]

32. 16 or 31 [Diabetes or Diet or Exercise]

33. self care/ or blood glucose self-monitoring/ or self administration/ or self medication/

34. (self adj3 (help or care* or manage* or administer* or monitor* or medicat* or treat* or inject*)).tw.

35. (goal* adj3 set*).tw.

36. exp Motivation/

37. Patient Education as Topic/

38. *health promotion/mt

39. exp computer-assisted instruction/

40. exp teaching materials/

41. ((patient or patients) adj5 (information or leaflet* or education* or educate* or book* or pamphlet* or resource* or dvd* or cd or cds or "cd rom*" or multimedia or web* or internet*)).tw.

42. (education adj2 (program* or session*)).tw.

43. ((skill or skills) adj2 teach*).tw.

44. self-help devices/

45. (("self help" or "self care" or "self manage*") adj5 (device* or tool* or technolog*)).tw.

46. (assistive adj2 technolog*).tw.

47. or/33-46 [Self Care Interventions]

48. clinical trial/ or clinical trial, phase i/ or clinical trial, phase ii/ or clinical trial, phase iii/ or clinical trial, phase iv/ or controlled clinical trial/ or multicenter study/ or randomized controlled trial/

49. exp Clinical Trials as Topic/

50. Evaluation studies/

51. Validation studies/

52. research design/ or cross-over studies/ or double-blind method/ or matched-pair analysis/ or random allocation/ or "reproducibility of results"/ or sample size/ or exp "sensitivity and specificity"/ or single-blind method/ or Early Termination of Clinical Trials/

53. (pre post or pre test or post test or non ramdomi?ed or quasi experiment).tw.

54. Feasibility studies/

55. Intervention studies/

56. Pilot projects/

57. exp program evaluation/

58. placebo*.tw.

59. (random* adj3 (study or studies or trial or trials)).tw.

60. (random* adj3 (allocation or assign* or allocate*)).tw.

61. (study adj (pilot or feasibility or evaluation or validation)).tw.

62. (studies adj (pilot or feasibility or evaluation or validation)).tw.

63. ((blind or mask*) adj2 (singl* or doubl* or trebl* or tripl*)).tw.

64. (control adj group*).tw.

65. ("outcome study" or "outcome studies" or quasiexperimental or "quasi experimental" or quasi-experimental or "pseudo experimental").tw.

66. case reports/

67. (crossover* or "cross over*" or cross-over*).tw.

68. ((trial or trials) adj2 (clinical or controlled or cluster or factorial)).tw.

69. case stud*.tw.

70. intervention.tw.

71. or/48-70 [Intervention Studies]

72. medication adherence/

73. patient compliance/

74. (adher* or nonadher* or non-adher*).tw.

75. (compliance or noncompliance or non-compliance).tw.

76. exp Attitude to Health/

77. "Patient Acceptance of Health Care"/ or Treatment Refusal/ or Patient Participation/ or exp Patient Satisfaction/ or Patient Dropouts/

78. or/72-77 [Adherence search]

79. 71 or 78 [Intervention or Adherence studies]

80. 9 and 32 and 47 and 79

81. exp animals/ not (exp animals/ and exp humans/)

82. (adolescent/ or exp child/ or exp infant/) not ((adolescent/ or exp child/ or exp infant/) and exp adult/)

83. dementia.tw.

84. exp dementia/

85. or/81-84

86. 80 not 85

**Database: Ovid MEDLINE(R) In-Process & Other Non-Indexed Citations <July 14, 2015>**

1 ((Development* or Learning or intellectual*) adj3 (disab* or disorder* or disturbance* or impair* or difficulty or difficulties)).tw. (5297)

2 ((Subnormal* or below normal) adj3 (intellect* or mental* or learning or IQ)).tw. (19)

3 (Mental* adj3 (disab* or impair* or deficien* or retard* or handicap*)).tw. (1684)

4 (cogniti* adj5 (disab* or disorder* or disturbance* or impair* or difficulty or difficulties)).tw. (7269)

5 or/1-4 [Learning Disabilities] (13569)

6 (diabet* adj3 (ii or "type 2" or two)).tw. (12211)

7 (diabet* adj3 ("non insulin dependent" or stable or "adult onset" or "maturity onset" or "ketosis reistant" or "slow onset")).tw. (328)

8 niddm.tw. (123)

9 mody.tw. (67)

10 ((diet* or eat or eating) adj2 (behav* or habit* or therap* or treatment* or intervention*)).tw. (4353)

11 (weight adj2 (loss* or control or gain* or reduc*)).tw. (11801)

12 (physical adj2 (exercis* or activit*)).tw. (9312)

13 exercise*.ti. (6677)

14 or/6-13 [Diabetes or diet or execise] (39817)

15 (self adj3 (help or care* or manage* or administer* or monitor* or medicat* or treat* or inject*)).tw. (7821)

16 (goal* adj3 set*).tw. (628)

17 ((patient or patients) adj5 (information or leaflet* or education* or educate* or book* or pamphlet* or resource* or dvd* or cd or cds or "cd rom*" or multimedia or web* or internet*)).tw. (9456)

18 (education adj2 (program* or session*)).tw. (2260)

19 ((skill or skills) adj2 teach*).tw. (275)

20 (("self help" or "self care" or "self manage*") adj5 (device* or tool* or technolog*)).tw. (117)

21 (assistive adj2 technolog*).tw. (126)

22 or/15-21 [Self Care Interventions] (19508)

23 placebo*.tw. (11341)

24 (random* adj3 (study or studies or trial or trials)).tw. (30661)

25 (random* adj3 (allocation or assign* or allocate*)).tw. (9782)

26 (study adj (pilot or feasibility or evaluation or validation)).tw. (109)

27 (studies adj (pilot or feasibility or evaluation or validation)).tw. (38)

28 ((blind or mask*) adj2 (singl* or doubl* or trebl* or tripl*)).tw. (7578)

29 (control adj group*).tw. (30204)

30 ("outcome study" or "outcome studies" or quasiexperimental or "quasi experimental" or quasi-experimental or "pseudo experimental").tw. (1572)

31 (crossover* or "cross over*" or cross-over*).tw. (7194)

32 ((trial or trials) adj2 (clinical or controlled or cluster or factorial)).tw. (39188)

33 case stud*.tw. (7878)

34 intervention.tw. (41508)

35 (adher* or nonadher* or non-adher*).tw. (12536)

36 (compliance or noncompliance or non-compliance).tw. (7256)

37 or/23-36 [Intervention or Adherence Studies] (143424)

38 5 and 14 and 22 and 37 (12)

39 dementia.tw. (6585)

40 38 not 39 (10)

**Database: PsycINFO <1806 to July Week 1 2015>**

1 learning disorders/ or learning disabilities/ (21529)

2 exp Intellectual Development Disorder/ (40106)

3 reading disabilities/ (5029)

4 developmental disabilities/ (10701)

5 cognitive impairment/ (25859)

6 ((Development* or Learning or intellectual*) adj3 (disab* or disorder* or disturbance* or impair* or difficulty or difficulties)).tw. (67254)

7 ((Subnormal* or below normal) adj3 (intellect* or mental* or learning or IQ)).tw. (468)

8 (Mental* adj3 (disab* or impair* or deficien* or retard* or handicap*)).tw. (47379)

9 (cogniti* adj5 (disab* or disorder* or disturbance* or impair* or difficulty or difficulties)).tw. (49087)

10 or/1-9 [Learning Disabilities] (169149)

11 self care skills/ (3611)

12 skill learning/ (3924)

13 self management/ or self help techniques/ or self instructional training/ (8655)

14 health care seeking behavior/ (3144)

15 self referral/ (190)

16 drug self administration/ (1627)

17 self monitoring/ (2587)

18 client education/ or health knowledge/ or health literacy/ (10144)

19 (self adj3 (help or care* or manage* or administer* or monitor* or medicat* or treat* or inject*)).tw. (49499)

20 ((skill or skills) adj2 teach*).tw. (5040)

21 (education adj2 (program* or session*)).tw. (19703)

22 instructional media/ or educational audiovisual aids/ or reading materials/ (4041)

23 goal setting/ or goals/ or motivation/ (51670)

24 (goal* adj3 set*).tw. (8036)

25 *health promotion/ (14428)

26 ((patient or patients or client*) adj5 (information or leaflet* or education* or educate* or book* or pamphlet* or resource* or dvd* or cd or cds or "cd rom*" or multimedia or web* or internet*)).tw. (25213)

27 (("self help" or "self care" or "self manage*") adj5 (device* or tool* or technolog*)).tw. (341)

28 (assistive adj2 technolog*).tw. (1232)

29 or/11-28 [Self Care Interventions] (186568)

30 diabetes mellitus/ (4196)

31 (diabet* adj3 (ii or "type 2" or two)).tw. (5251)

32 (diabet* adj3 ("non insulin dependent" or stable or "adult onset" or "maturity onset" or "ketosis reistant" or "slow onset")).tw. (251)

33 (niddm or mody).tw. (121)

34 exp diets/ (9876)

35 exp eating behavior/ (15000)

36 ((diet* or eat or eating) adj2 (behav* or habit* or therap* or treatment* or intervention*)).tw. (14179)

37 (weight adj2 (loss* or control or gain* or reduc*)).tw. (19556)

38 obesity/ (17573)

39 weight loss/ or weight control/ (5650)

40 (physical adj2 (exercise* or activit*)).tw. (24976)

41 exercise*.ti. (11205)

42 physical activity/ or exp exercise/ (27278)

43 or/30-42 [Diabetes or Diet or exercise] (95466)

44 exp clinical trials/ or experimental design/ (18329)

45 exp treatment effectiveness evaluation/ (18683)

46 exp mental health program evaluation/ (1929)

47 exp random sampling/ (654)

48 randomi*.tw. (55495)

49 (crossover or cross-over).tw. (7703)

50 exp placebo/ (4140)

51 placebo*.tw. (33161)

52 evaluation/ or exp program evaluation/ (33628)

53 exp test construction/ (86622)

54 intervention/ or early intervention/ (50740)

55 (random* adj3 (study or studies or trial or trials)).tw. (39628)

56 (random* adj3 (allocation or assign* or allocate*)).tw. (32680)

57 (study adj (pilot or feasibility or evaluation or validation)).tw. (250)

58 (studies adj (pilot or feasibility or evaluation or validation)).tw. (70)

59 (control adj group*).tw. (62302)

60 ("outcome study" or "outcome studies" or quasiexperimental or "quasi experimental" or quasi-experimental or "pseudo experimental").tw. (11473)

61 case report/ (22563)

62 case stud*.tw. (74183)

63 ((trial or trials) adj2 (clinical or controlled or cluster or factorial)).tw. (47610)

64 intervention.tw. (171051)

65 or/44-64 [Intervention studies] (531136)

66 treatment compliance/ (11882)

67 Compliance/ (3700)

68 (adher* or nonadher* or non-adher*).tw. (25814)

69 (compliance or noncompliance or non-compliance).tw. (21383)

70 Client Attitudes/ or Client participation/ or Treatment Barriers/ or Treatment Dropouts/ or Treatment Refusal/ (20843)

71 or/66-70 [Adherence studies] (66948)

72 65 or 71 (581333)

73 10 and 29 and 43 and 72 (125)

74 dementia.tw. (48484)

75 exp dementia/ (57443)

76 74 or 75 (69814)

77 73 not 76 (116)
