# Supplementary material for: Measurement of adherence in a randomised controlled trial of a complex intervention: supported self-management for adults with learning disability and type 2 diabetes
Source: BMC Med Res Methodol. 2016 Oct 6;16:132. doi: 10.1186/s12874-016-0236-x (PMC5052902; doi:10.1186/s12874-016-0236-x)
Supplement: Additional file 2: — Data Extraction Form. (DOCX 19 kb) [file 12874_2016_236_MOESM2_ESM.docx]

**Additional file 2 Data Extraction Form**

**Reviewer:**

**1. Authors:**

**2. Title:**

**3. Country:**

**4. Journal & Year:**

**5. Summary of trial**

**6. Study design**

**7. Study participants**

**8. Interventions**

|  | Description | Provided by | Delivered where |
| --- | --- | --- | --- |
| Self-management intervention |  |  |  |
| Control |  |  |  |
| Other |  |  |  |

**9. Content of self-management intervention. Did it include…**

|  | **Item** | **Yes / No** | **Details** |
| --- | --- | --- | --- |
| 1 | Shopping and choosing food |  |  |
| 2 | Preparing food (cooking, portions) |  |  |
| 3 | Eating behaviours |  |  |
| 4 | Informal, day-to-day activity |  |  |
| 5 | Formal activity - e.g. specific exercise class |  |  |
| 6 | Remembering tablets / medication |  |  |
| 7 | Smoking |  |  |
| 8 | Alcohol |  |  |
| 9 | Foot care |  |  |
| 10 | Other |  |  |

**10. How was content delivered?**

|  | **Component** | **Details** | **Directed at whom** |
| --- | --- | --- | --- |
| 1 | Educational materials |  |  |
| 2 | Educational sessions |  |  |
| 3 | Self-monitoring resources |  |  |
| 4 | Use of prompts |  |  |
| 5 | Promoting use of services – e.g. cheaper swimming pool sessions |  |  |
| 6 | Individual goal setting |  |  |
| 7 | Action plans (e.g. doing exercise on own) |  |  |
| 8 | Attendance at peer support groups |  |  |
| 9 | Attendance at statutory groups |  |  |
| 10 | Skills training (e.g. problem solving, assertiveness / social skills) |  |  |
| 11 | Managing emotions |  |  |
| 12 | Other |  |  |
| 13 | Other |  |  |
| 14 | Other |  |  |
| 15 | Other |  |  |

**11. Was adherence measured? Yes / No**

| **Comments** |
| --- |

**12. Measured for all or a sample of participants / providers?**

| 1 | All |  |
| --- | --- | --- |
| 2 | Sample (how defined, proportion of sample, justification – and source of justification) |  |

**13. What did they measure (in terms of provider adherence)?**

|  | **Component** | **Did they measure this? (Y/N/NA)** | **What was described as being measured?** | **What was reported?** | **Was this incorporated into the analysis?** |
| --- | --- | --- | --- | --- | --- |
| **To ensure treatment integrity** | | | |  |  |
| 1 | Who delivered the intervention (credentials, requirements) |  |  |  |  |
| 2 | Training of provider |  |  |  |  |
| 3 | Competence of provider |  |  |  |  |
| 4 | Supervision of provider |  |  |  |  |
| **To ensure actual delivery** | | | |  |  |
| 5 | Delivery of educational materials |  |  |  |  |
| 6 | Provision of educational sessions |  |  |  |  |
| 7 | Provision of / training in self-monitoring resources |  |  |  |  |
| 8 | Training in use of prompts |  |  |  |  |
| 9 | Promoting use of services – e.g. cheaper swimming pool sessions |  |  |  |  |
| 10 | Individual goal setting |  |  |  |  |
| 11 | Providing action plans (e.g. doing exercise on own) |  |  |  |  |
| 12 | Peer support groups |  |  |  |  |
| 13 | Statutory groups |  |  |  |  |
| 14 | Provision of skills training (e.g. problem solving, assertiveness / social skills) |  |  |  |  |
| 15 | Training re: managing emotions |  |  |  |  |
| 16 | Other |  |  |  |  |

**14. What did they measure (in terms of user adherence)?**

|  | **Component** | **Did they measure this? (Y/N/NA)** | **What was described as being measured?** | **What was reported?** | **Was this incorporated into the analysis?** |
| --- | --- | --- | --- | --- | --- |
| 1 | Receipt and use of educational materials |  |  |  |  |
| 2 | Attendance at educational sessions |  |  |  |  |
| 3 | Use of self-monitoring resources |  |  |  |  |
| 4 | Use of prompts |  |  |  |  |
| 5 | Use of promoted services – e.g. cheaper swimming pool sessions |  |  |  |  |
| 6 | Individual goal setting |  |  |  |  |
| 7 | Action plans (e.g. doing exercise on own) |  |  |  |  |
| 8 | Attendance at peer support groups |  |  |  |  |
| 9 | Attendance at statutory groups |  |  |  |  |
| 10 | Skills training (e.g. problem solving, assertiveness / social skills) |  |  |  |  |
| 11 | Managing emotions |  |  |  |  |
| 12 | Other |  |  |  |  |

**15. How did they score and analyse their measure(s)**

| 1 | Standard questionnaire scoring / analysis |  |
| --- | --- | --- |
| 2 | Adherence checklist scored according to trial ‘rules’ |  |
| 3 | Single scores |  |
| 4 | Modelling? |  |
| 5 | Other |  |
