# Supplementary material for: Measurement of adherence in a randomised controlled trial of a complex intervention: supported self-management for adults with learning disability and type 2 diabetes
Source: BMC Med Res Methodol. 2016 Oct 6;16:132. doi: 10.1186/s12874-016-0236-x (PMC5052902; doi:10.1186/s12874-016-0236-x)
Supplement: Additional file 3: — Adherence Checklist – developed from the data extraction form. (DOCX 16 kb) [file 12874_2016_236_MOESM3_ESM.docx]

Additional file 3 **Adherence Checklist – developed from the data extraction form**

| **Elements** | **Considerations for describing, monitoring and reporting each element** | | | | **Considerations for adherence measurement** |
| --- | --- | --- | --- | --- | --- |
| General intervention details | What type of intervention (e.g. therapy vs. self-management) | Where is it provided | Who provides it (and do they need training, supervision) | Who is it delivered to (population +/- supporter) | Measurement of provider credentials, training, supervision, feedback, competence. Consider all those in receipt. |
| Content of the intervention | e.g. activity classes, learning how to choose the right foods, stopping smoking | | | | Evidence that content was delivered as intended |
| How the content is delivered | Technique (e.g. formal education, goal setting) and method (e.g. individual / group sessions, provision of written materials) | | | |  |
| Receipt and use of the intervention | e.g. receipt of materials, attendance at sessions, carrying out self-management tasks, changing diet | | | | Evidence of receipt (e.g. log of attendance, receipt of participant diaries) |
