# Supplementary material for: Measurement of adherence in a randomised controlled trial of a complex intervention: supported self-management for adults with learning disability and type 2 diabetes
Source: BMC Med Res Methodol. 2016 Oct 6;16:132. doi: 10.1186/s12874-016-0236-x (PMC5052902; doi:10.1186/s12874-016-0236-x)
Supplement: Additional file 4: — Summary of Papers Reviewed. This table summarises the study design, intervention content, techniques employed, format of intervention delivery, and steps taken to ensure provider and participant adherence for all papers meeting eligibility criteria and reviewed as part of this work. (DOCX 41 kb) [file 12874_2016_236_MOESM4_ESM.docx]

**Summary of papers reviewed**

| **Authors** | **Study summary**  (design, participants, intervention) | **Intervention content**  (topics / components) | **Techniques employed & format of delivery** | **Ensuring provider adherence** | | **Measuring actual adherence to the intervention**  (provider and participant adherence) | |
| --- | --- | --- | --- | --- | --- | --- | --- |
|  |  |  |  | **Steps to ensure adherence** | **Measurement of those steps** | **What was described as being measured** | **What was reported** |
| Aronow & Hahn (2005) | Pilot study including 201 adults with IDD.  Two groups assigned to one of two in-home health interventions. | 1. Advanced Practice Nurse (APN) intervention.  *The APN intervention is described in detail in the companion*  *article (Hahn & Aronow 2005) below.*  2. Health Risk Appraisal | Participants from private residences or group homes. Interviewed by Nurse Practitioner.  1. Educational materials  Action plans.  Reinforcement  Promoting use of services  2. Educational materials | - | - | Provision of educational materials. | Interview compliance. |
| Bazzano et al (2009) | Single group pilot study of a community-based health promotion intervention for adults with LD who were overweight and had one other risk factor for diabetes / metabolic syndrome. 68 participated, 44 completed.  Intervention: Healthy Lifestyle Change Program (HLCP). Twice weekly 2-hour sessions for 7 months, delivered by professionals and peer mentors, including education and exercise. | Shopping and choosing food  Preparing food  Eating behaviour  Informal activity  Medications  Self-care  Behaviour modification  Supervised physical activity | Didactic elements delivered by professionals with expertise in LD, with assistance from peer mentors  Delivered at a community organisation  One to one health management education  Educational materials  Promoting use of services (accessing healthcare) | Provider background.  Peer mentor training. |  |  | Attrition rate, completers, attendance rate range for ‘completers’, mean and median attendance rates. |
| Bergstrom et al. (2013)  *(results from Elinder 2010 paper)* | Cluster RCT for adults with mild-moderate LD who live in community residences, as well as staff within the residences.  130 participants.  30 residences.  Intervention: Materials for staff (Focus Health), peer group meetings for staff, training for health ambassadors in each residence, health education sessions for residents. | Health education for residents (health issues, healthy foods, physical activity).  Health ambassador meetings and coaching  Study circle for staff (peer education) | Health educator delivers educational sessions to residents within community residences.  Staff meet (study circle) within the residences.  Health ambassadors from various residences meet externally (network meetings). | Training and coaching of health ambassadors and staff. |  | Health ambassador attendance at network meetings (score)  Staff circle – number of sessions held (score).  Residents’ health course – number of sessions held (score).  Fidelity score for each residence based on the above. | Median values of fidelity scores. Divide homes into two groups – low and high fidelity. |
| Bodde et al (2011) | Pilot study developing and field testing the intervention in 42 adults with mild-moderate LD.  Intervention: Promoting Health Through Physical Activity, Knowledge and Skills (PHPAKS) | Healthy eating  Physical activity & safety | Delivered by an instructor at a disability day service centre.  Educational materials  Educational sessions  Promoting use of services  Skills training | Script to follow for each lesson. | Researcher observation of 4 random sessions to assess adherence to the script. | Non-attendance. |  |
| Bodde et al (2012) | Single group pre-post delayed treatment study of PHPAKS intervention (described above).  Included 42 adults with mild-moderate LD. | As above | Delivered at two agencies serving adults with LD. | *-* | *-* | *-* | Overall drop-out rates reported. |
| Bradley (2005) | Single group (n=9) study evaluating the effect of healthy eating sessions on weight loss for women with LD. | Shopping and choosing food  Preparing food | Group educational sessions  Delivered by Dietician, in the community. | Dietician employed to deliver sessions | - | Attendance.  Completion of food diary | No. sessions delivered overall.  ‘Excellent attendance’. |
| Chapman, Craven & Chadwick (2005) | Pre-post test (non-randomised) study evaluating the effect of health practitioner input vs. no input on BMI of adults with LD. | Physical activity (advice, planning, activity program)  Diet  Health | Home visits by Healthy Living Co-ordinator (a physiotherapist).  Educational materials.  Action plans.  Promoting use of services. | - | - | - | - |
| Elinder et al (2010) | Protocol paper for a cluster RCT for adults with mild-moderate LD who live in community residences, as well as staff within the residences.  Intervention: Materials for staff (Focus Health), peer group meetings for staff, training for health ambassadors in each residence, health education sessions for residents. | Health education for residents.  Health ambassador meetings and coaching  Study circle for staff (peer education) | Health educator delivers educational sessions to residents within the residences.  Staff meet (study circle) within the residences.  Health ambassadors from various residences meet externally. | Training and coaching of health ambassadors and staff. | Frequency of meetings.  Adherence score for each residence (observation of themes covered in sessions). | Attendance counts for residents, staff and health ambassadors  Adherence score (action plans set) | *No results reported as this is a protocol paper* |
| Ewing et al (2004) | Evaluation of the Health Education Learning Program (HELP) in two groups of adults: those with mild-moderate LD (N=92) and ‘normal learners’ (N=97), all with BMI > 27.  Intervention: 8 x weekly sessions, targeting a specific issue each week. | Shopping and choosing food  Eating behaviour  Informal activity  Self-care (relapse prevention) | Delivered in a Primary Care Centre by two Health Educators.  Educational sessions  Skills training (communication)  Home visits (grocery store visit, dietary plan, exercise plan)  Goal setting  Action plans | Regular supervision  Qualifications of health educators | Observed for adherence to the curriculum | - | Attrition, no. home visits, nos. attending 4+ classes overall and by group |
| Fisher (1986) | Behavioural self-control vs. behavioural self-control + physical activity. N=17 overweight female adults with mild-moderate LD.  Intervention: Behavioural control (as described by Rotatori & Fox 1981) | Physical activity: daily walking.  Eating behaviour (including reinforcement) | Delivered at a sheltered workshop by a ‘group leader’.  Educational sessions.  Exercise sessions. | - | - | Cooperation rating scale.  Homework assignments | - |
| Fox, Rosenberg, Rotatori (1985) | 15 adults with moderate LD and obesity, employed in a sheltered workshop and living with parents.  Intervention: Behavioural treatment program designed to promote weight loss.  8 participants were included in the ‘parent involvement’ group, 7 in the subject-only group. Pre-post test measures. | Eating behaviours  Self-reinforcement patterns.  Parent involvement group:  Support, encouragement, reward  Group rewards provided. | Twice-weekly educational sessions for 10 weeks for both groups.  3 monthly maintenance meetings thereafter. | - | - | Phone calls to parents to check understanding and that reward was given.  Subjects questioned re: receipt of parental reward.  Daily homework.  Attendance at sessions. | The text reports that parents were consistent in providing a reward.  The text reports that no. of homework forms completed was recorded. |
| Hahn & Aronow (2005) | Description of the intervention detailed in Aronow & Hahn 2005 (above) | Physical activity.  Choosing food.  Referral to services (based on assessed health needs). | Advanced Practice Nurse visited participants at home to assess needs.  Educational materials (tailored to individual).  Action plans.  Promoting use of services. | APNs had specific qualifications.  Followed standard guidance. | No. of recommendations made (overall) is reported. | APNs rated adherence (scale of 0=none, 1=partial & 2=complete) to recommendations at each visit. | Report percentage adherence to recommendations.  Report mean no. visits made by APN.  Report at least 1/3 recommendations were for self-care activities. |
| Heller (2004) | Adults with Downs Syndrome randomised to receive either a fitness and health education program (N=32) or control (N=21). | Formal activity  Choosing food  Eating behaviours | Group exercise sessions  Group educational sessions  Educational materials  Skills training (self-efficacy)  Goal setting  Action plans | - | - | - | No. completing pre- and post-assessments reported. |
| Humphries et al (2008) | Nutrition and education support program in 4 community-based homes for adults with ID. Included adults with ID (32), service staff (20) and home managers (4).  Intervention: MENU-AIDDS program in each home | Shopping / choosing food (food group charts, shopping organisers)  Preparing food (menus, recipes) | Delivered in each home by health specialists attached to the home – to residents and to staff.  Educational materials  Educational sessions | Training of Health Specialists  Delivery of training to homes |  | Interviews with home managers & staff re: use of materials | Degree to which materials were used is coded on a 3 point scale from interview data |
| Jones et al (2006) | Single group pre-post study. N=22 participants with severe LD and challenging behaviour / profound LD & other physical disabilities.  Intervention: 16 week individualised exercise program to improve goal attainment. | Formal activity | Two nursing assistants as program co-ordinators.  Exercise sessions held at NHS location.  Goal setting. | - | - | Individual goal setting by provider  Level of participation, enjoyment and duration of session for each participant | Duration of sessions for some participants |
| Lunsky, Straiko, Armstrong (2003) | Single group study (n=22). 8 week curriculum (‘women be healthy’) for women with mild-moderate ID. | Health education (exercise, medical examination, diet, hygiene)  Stress reduction  Exposure to the medical setting | Group educational sessions at a clinic – delivered by two female co-leaders  Educational visits to medical facility  Skills training (coping strategies, assertiveness) | Leaders had extensive experience with this population | - | Attendance  Homework | Attendance was ‘not as consistent as the leaders would have liked’ |
| McCarren & Andrasik (1990) | Two group study including 12 adults from a cerebral palsy centre.  Intervention: 19 week behavioural weight loss package for both groups, with one group additionally including caretaker / parent contact with the diet leader. | Eating behaviour  Physical activity | Delivered by a graduate (diet leader) and undergraduate (assistant) in a cerebral palsy centre.  Educational materials sent to carer.  Educational sessions  Goal setting | - | - | Self-monitoring (eating habit records) | Drop out. |
| McDermott et al (2012) | RCT of ‘Steps To Your Health’ (STYH) vs. non-interventional group classes (focus on hygiene and safety). Adapted from HELP (Ewing et al 2004).  N=443 community dwelling adults with mild-moderate LD.  STYH: health promotion intervention targeting specific health issues in 8 group educational sessions. | Shopping & choosing food  Preparing food  Eating behaviours  Day to day activity | Group educational sessions delivered by a health educator in local disability agency service facilities. | Experience of health educator | - | - | Participant retention at follow-up time points. |
| Mann (2006) | Health promotion program for 192 overweight and obese adults with LD living in independent or supported setting. Self-selecting / voluntary participation.  Intervention: ‘Steps To Your Health’ (as above – McDermott et al 2012) | Exercise  Choosing food  Stress reduction | Group educational sessions, taught by staff members employed by service providers. | Training and technical support provided by two university professionals. |  | Attendance | Retention (drop out at follow-up) |
| Marks B et al. (2013) | Randomised study of a 12 week exercise and health education programme vs. control group. Both staff (N=34) and adults with mild-moderate Intellectual Disability (N=67) recruited. | Formal exercise  Shopping and Choosing food  Preparing food | Sessions run by staff participants (various professions) in their respective community based organisations (running day programmes).  Educational sessions and physical activity sessions. | 8 hour workshop attended by staff and run by the Principal Investigator and project co-ordinator | - | - | Completion of the course (report no. participants who did not complete all required sessions). Those completing less than 30/36 sessions were removed from the analysis. |
| Marshall, McConkey & Moore (2003) | Health promotion classes in the NHS for people with LD who are overweight.  Pre-post measurements of weight. | Adapted from the ‘Activate’ program materials produced by the Health Promotion Agency in Northern Ireland.  Healthy eating  Physical activity | Educational group sessions  Delivered by 2 Learning Disability Nurses in 3 group settings (2 within day centre / facility, 1 in a leisure centre). | 1 nurse attended a 10 session training course | - | - | Report that ‘only one person dropped out’.  Two groups ran for 6 weeks, and one for 8 weeks. |
| Mate-Kole, Danquah, Twum, Danquah (1999) | 15 adults with moderate LD and behavioural problems.  Evaluation of 3 behavioural interventions (‘differential reinforcement of other behaviours’ vs. ‘mutual goal setting’ vs. routine care)  Pre-post design. | Individualised self-care behaviours (e.g. dressing, bathing, feeding) | Delivered within a rehabilitation centre, by staff working in the centre.  Goal setting.  Behavioural reinforcement. | Staff type & training described. | Report that there was no staff attrition. | Staff met with participants at predetermined times to develop & evaluate rehabilitation plans. | - |
| Pett (2013) | Healthy lifestyle intervention for obese, home dwelling young adults (YA) with mild – moderate IDD, including primary care givers (CG). 3 cohorts: 1. YA only (N=12), 2. YA + CG (N=11), 3. CG only (N=8). 1 & 2 randomly assigned. 2 acted as wait list control for 1. | YA components:  Healthy eating  Formal activity  Informal activity  Stress reduction  CG components:  Healthy behaviours  Healthy environment | For YAs, sessions delivered by trained personnel from / at the recreation centre, as well as volunteers from the university and community.  Education sessions  Skills training / stress management  For CGs, education sessions facilitated by dietician, social worker, and students. | - | CG sessions recorded ‘to ensure treatment adherence’ | - | Attendance (> 70% for those included in analysis) |
| Poyner (2008) | Single group pilot project including 19 participants with LD and their support workers (n=7).  Intervention: ‘steps to fitness’. | Formal activity  Shopping and choosing food  Preparing food | At local gym:  Group activity sessions  Group educational sessions  Promoting use of gym | Appropriate professionals delivered sessions (e.g. gym instructor, dietician, nurse specialist) | - | Attendance  Homework | Staff variation in attendance noted.  Homework completion and attendance by people with LD linked to degree of support worker participation in sessions.  Drop out reported. |
| Rotatori, Fox, Switzky (1979) | Six overweight adolescents with Down’s syndrome & moderate LD participated in a parent-teacher administered behavioural weight loss program. Treatment and maintenance lasted for 19 weeks. | Delivered to parent & teacher: helping acquire new habits, monitoring progress, providing rewards.  Delivered to subjects:  Physical activity.  Eating behaviours.  Techniques for weight loss.  Self-monitoring techniques.  Reinforcement / rewards. | Parent / teacher:  Skills training.  Subjects:  Educational sessions with classroom teacher (3 per week for 14 weeks, then 5 maintenance sessions).  Role play & feedback.  Educational materials. | Parents and teachers attended presentation | - | Food diary  Behaviour checklist  Parents’ progress notes  Phone calls from teacher to parent | - |
| Rotatori & Fox (1980) | 30 adolescents with moderate LD, living with parents, and overweight, randomly assigned to behavioural therapy (n=12) vs. social-nutrition (n=12) vs. wait list (n=6).  Parents were involved in intervention components. | Behavioural therapy  Eating behaviours  Techniques for weight loss (subjects and parents).  Reinforcement at the end of each week when weight lost.  Social nutrition  Discussion of topics relating to food and exercise | Behavioural therapy Educational sessions (three times weekly for 14 weeks).  Follow-up phone calls with parent.  5 x weekly maintenance group sessions at end of educational phase (videos of techniques learned and forms completed).  Social nutrition  Group meetings with school nurse ~4 times / week.  Goal setting (weight)  Managing emotions. | - | - | Parents’ progress notes re: use of techniques at home.  Phone calls to parent by teacher to check progress / understanding.  Daily homework (behaviour checklist, food diary, weight record) | - |
| Rotatori (1980) | N=18 volunteer adults with LD residing in a community living facility were randomised to behavioural weight reduction program vs. control. | Eating behaviours  Preparing food  Informal activity | Intervention delivered by first author.  Educational sessions  Skills training (managing emotional responses)  Goal setting  Action plans. | - | - | Daily weight records  Food diaries  Reimbursement for weight loss and handing in ‘homework’ | - |
| Rotatori et al (1986) | N=13 overweight adults with mild-mod LD. All exposed to behavioural weight reduction program (12 weeks) followed by 10 months weekly weigh in. Then randomly allocated to booster session group vs. treatment maintenance group (both 12 months). | Booster sessions included:  Eating behaviours  Preparing & choosing food  Informal activity  Both groups:  Weigh in and weight loss recognition | Group sessions at vocational training centre directed by school nurse  Educational sessions  Educational materials  Self-monitoring resources  Letters and phone calls from diet leaders. | - | - | Work books  Weekly meal and exercise records | - |
| Sailer (2006) | Evaluation of a weight loss program for obese individuals with mild mental retardation, living independently in the community. N=6.  (Based on Rotatori & Fox 1984) | Eating behaviours  Choosing and preparing food  Informal activity | Delivered by a group leader at the human services centre.  Education sessions (including modelling and rehearsing behaviours)  Educational materials  Goal setting and rewards  Phone calls by therapist between sessions. | Same group leader for both groups (identical group content, just split for practical reasons) | - | Homework set and reviewed each session  Food diary  Attendance rewarded. | Report attendance rates for some of the individual participants  One participant reported as filling in the homework at the start of each session – discuss reliability. |
| Wilhite et al (2012) | Single group pre-post study including 15 participants with LD and 10 carers.  Intervention: Get FIT (Fitness, Integration, Training) program. 3 individual nutrition consultations, 12 weeks’ exercise sessions. | Choosing food  Formal activity | Delivered by undergraduate students at a university  Educational materials  Educational sessions  Exercise sessions  Goal setting & action plans (exercise and diet) | Training of students  Review of process notes and feedback from course instructor | Process notes written by students (providers).  Diet sheets provided to participant and supervisor. | Self-monitoring (food log) | Report number of completers. |
