# Supplementary material for: Measurement of adherence in a randomised controlled trial of a complex intervention: supported self-management for adults with learning disability and type 2 diabetes
Source: BMC Med Res Methodol. 2016 Oct 6;16:132. doi: 10.1186/s12874-016-0236-x (PMC5052902; doi:10.1186/s12874-016-0236-x)
Supplement: Additional file 5 — Case Report Forms developed to collect adherence data for the OK Diabetes Trial. (PDF 447 kb) [file 12874_2016_236_MOESM5_ESM.pdf]

OK-Diabetes

FORM 48

Phase II Session Record

Person Initials		Date of Birth	Day Month Year	Phase I ID	

To be completed by the Study Nurse delivering the Self-Management Intervention
(Only to be completed for sessions involving some elements of intervention delivery, not those which are purely administrative – e.g. arranging appointments.)

Section A – Session Attendance

Planned date of session: Day Month Year **or tick if** ☐ No sessions could be arranged (Please complete Section D)

Did this go ahead?
- ☐ Yes → Please skip to Section B
- ☐ No → Was another session arranged?
  - ☐ Yes → New date: Day Month Year (No further details required for this session)
  - ☐ No, did not want any further sessions → Please complete Sections C & D

Section B – Session Details

Length of session: Hours : Minutes

Session number:

Location of session:
- ☐ Person's home
- ☐ GP practice
- ☐ Day centre
- ☐ Other location, please specify

Who was present? *(Please tick yes/no/N/A for each)*

Person with diabetes *(study participant)*	☐ Yes	☐ No	
Consenting Supporter	☐ Yes	☐ No	☐ N/A – No consenting supporter
Other person/people	☐ Yes	☐ No	

If yes, who?
(Include details of who each person was and their role in relation to the person with diabetes)

What was the main focus of this session? *(Tick all that apply)*
- ☐ Getting started
- ☐ Setting goals
- ☐ Mapping support
- ☐ Checking progress

Did you refer them for a personal budget assessment? ☐ Yes ☐ No ☐ Already had

Session 1 only: Did you give them the standard leaflet? ☐ Yes ☐ No

Was this the last session?
- ☐ Yes → Please complete Sections C & D
- ☐ No → No further details required – Please sign & date this form and return to CTRU

Completed by: Date: Day Month Year

Form continues on next page ►►

Prior to returning this form to CTRU you must make a copy of the form and any amendments for retention at site. CTRU, University of Leeds (please see Investigator Site File for full contact details).

For office use only	Computerised		Verified/Checked	
	Date	Initials	Date	Initials

Person Initials		Date of Birth	Day Month Year	Phase I ID	

Section C – Engagement

To be completed after the last session

In your opinion:

1) How engaged was the person within the sessions?
   - ☐ Very engaged
   - ☐ Quite engaged
   - ☐ Not engaged – attention was not focussed

2) How engaged was the person with the materials?
   - ☐ Very engaged
   - ☐ Quite engaged
   - ☐ Not engaged – hardly used materials

3) How engaged was the consenting supporter with the sessions?
   - ☐ Very engaged
   - ☐ Quite engaged
   - ☐ Not engaged – attention was not focussed
   - ☐ Did not attend at all
   - ☐ N/A – no consenting Supporter

4) Was there anyone else who was engaged in the intervention implementation?
   - ☐ Yes → Who? 1. ______ 2. ______
   - ☐ No

Did you collect any materials at the last session?
- ☐ Yes → Please return these to CTRU via your supervisor
- ☐ No

Section D – Summary of Attendance

Please tick one of the following to describe the participant's attendance:

- ☐ Did not attend **any** sessions → Reason ______
- ☐ Did not attend **all** required sessions → Reason ______
- ☐ Completed all required sessions

Were more than 4 sessions attended?
- ☐ Yes → Reason ______
- ☐ No

Completed by ______ Date Day Month Year

Last Page ■

Prior to returning this form to CTRU you must make a copy of the form and any amendments for retention at site. CTRU, University of Leeds (please see Investigator Site File for full contact details).

For office use only	Computerised	Verified/Checked
	Date *Initials*	*Date* *Initials*

OK-Diabetes

FORM 49
Page 1 of 1

Phase II Intervention Delivery Details

Person Initials		Date of Birth	Day / Month / Year	Phase I ID	

To be completed by the independent reviewer from therapist notes and copies of completed charts

Section A – Session Details

Session date (Day / Month / Year) Session number

Nurse delivering intervention

What was covered during this session? *(Tick yes or no for each and give details as appropriate)*

Getting Started

	Yes	No	
1) My week timetable	☐	☐	
2) My life chart	☐	☐	
3) Looking after my diabetes	☐	☐	
4) 'I am going to' chart*	☐	☐	
5) 'What where when' chart	☐	☐	
6) Supporters & helpers checklist	☐	☐	*Nurse will have taken a photograph of these. Obtain from nurse records.*
7) 'I am going to' box*	☐	☐	**If yes**, what was/were the identified goal(s)? Choose a code from list below and specify if = 11 (Other): Goal 1 Code Description; Goal 2 Code Description — *Participant keeps these*
OR			
8) 'I am going to' plan*	☐	☐	**If yes**, what were the identified plans? Plan 1; Plan 2 — *Participant keeps these*
9) Supporter flashcard(s) given?*	☐	☐	**If yes**, how many? ☐ 1 ☐ 2 ☐ 3 ☐ 4<br>Were they: ☐ Left with the person to give to the supporter/helper ☐ Given to the supporter/helper in person ☐ Posted to the supporter/helper

Goal code list

1 = More fruit
2 = More vegetables
3 = Snack swaps (food)
4 = Snack swaps (fizzy drinks)
5 = Weight loss
6 = More physical activity
7 = Tablet taking
8 = Checking feet
9 = Retinal screening
10 = Seeing dentist
11 = Other (specify)

**May stay with participant, so nurse will have taken a photograph. Obtain from nurse records.*

Section B – Final Nurse Visit Only *(4 weeks after end of intervention delivery)*

Calendar sheets collected? ☐ Yes → How many? ☐ No

Section C – 6 Month Researcher Visit Only

Calendar sheets collected? ☐ Yes → How many? ☐ No

Completed by Date (Day / Month / Year) *Last Page* ■

Prior to returning this form to CTRU you must make a copy of the form and any amendments for retention at site. CTRU, University of Leeds (please see Investigator Site File for full contact details).

For office use only	Computerised — *Date* *Initials*	Verified/Checked — *Date* *Initials*

OK-Diabetes

FORM 50

Phase II Fidelity Scoring

Person Initials		Date of Birth	Day Month Year	Phase I ID	

To be completed by the independent reviewer once all relevant materials received

Fidelity Scoring – 4 Weeks Post-Randomisation

		Yes	No	Any	None
1.	Completed 'My Week Timetable' and 'My Life Chart'	☐	☐		
2.	Completed 'Looking after my Diabetes Chart'	☐	☐		
3.	Completed 'I am Going to Chart' and a 'What Where When Chart'	☐	☐		
4.	Has an 'I am Going to Box' and/or an 'I am Going to Plan'	☐	☐		
5.	Completed 'Supporters & Helpers Checklist'	☐	☐		
6.	A flashcard was provided for identified supporter and relevant helpers	☐	☐		
7.	Number of *unmarked* calendar sheets returned			☐	☐
8.	Number of *marked* calendar sheets returned			☐	☐
9.	The whole process was completed at least once	☐	☐		
10.	Evidence of repetition of process for further goals	☐	☐		

Fidelity Scoring – 6 Months Post-Randomisation

			Any	None
1.	Number of *unmarked* calendar sheets returned		☐	☐
2.	Number of *marked* calendar sheets returned		☐	☐

Comments and Notes

Note the dates between which calendar cards could have been completed

Completed by ______ Date Day Month Year

Last Page ■

Prior to returning this form to CTRU you must make a copy of the form and any amendments for retention at site.
CTRU, University of Leeds (please see Investigator Site File for full contact details).

For office use only	*Computerised* Date Initials	*Verified/Checked* Date Initials

OK-Diabetes

FORM 37

Supervision Details

To be completed by the Supervisor following supervision session with study nurse(s)

Name of supervisor

Name of nurse(s) in supervision session

Date of supervision session (Day Month Year)

Duration of supervision session (Hours : Minutes)

Method of supervision

- ☐ Face to face
- ☐ Telephone
- ☐ Email

Participants discussed

Phase I ID	Initials	Date of birth
		Day Month Year
		Day Month Year
		Day Month Year
		Day Month Year
		Day Month Year
		Day Month Year
		Day Month Year
		Day Month Year

Completed by

Date (Day Month Year)

Last Page ■

Prior to returning this form to CTRU you must make a copy of the form and any amendments for retention at site.
CTRU, University of Leeds (please see Investigator Site File for full contact details).

For office use only	*Computerised*		*Verified/Checked*	
	Date	*Initials*	*Date*	*Initials*
